# Sagittal anatomy in laparoscopic radical right hemicolectomy for right colon cancer based on membrane anatomy theory

**DOI:** 10.3389/fonc.2025.1614285

**Published:** 2025-07-31

**Authors:** Guofeng Pan, Weihong Zhang, Shiyu Huang, Jihuang Wu, Jian Chen, Jianbin Weng, Zipeng Zhu, Zhixing Guo, Yanchang Xu

**Affiliations:** ^1^ Department of Gastroenterological Surgery Unit 1, the Teaching Hospital of Putian First Hospital, Fujian Medical University, Putian, Fujian, China; ^2^ Department of gastroenterological surgery Unit 1, Putian First Hospital, The Affiliated Hospital of Putian University, Putian, Fujian, China; ^3^ Department of Gastroenterological Surgery, Institute of Minimally Invasive Surgery of Putian University, Putian, Fujian, China; ^4^ Department of operating room, the Teaching Hospital of Putian First Hospital, Fujian Medical University, Putian, Fujian, China

**Keywords:** right colon cancer, sagittal anatomy, membrane anatomy, anatomical approach, laparoscopy

## Abstract

**Objectives:**

This study aimed to explore the concept of sagittal anatomy in laparoscopic radical right hemicolectomy through the lens of membrane anatomy.

**Methods:**

A retrospective study reviewed clinical records of 128 patients with right colon cancer who received laparoscopic radical right hemicolectomy at the Department of Gastrointestinal Surgery Unit 1, The First Hospital of Putian City, Fujian Province, between December 2020 and December 2022. Among the participants, 70 were male and 58 were female, with an average age of 62 years. All patients received standardized laparoscopic radical right hemicolectomy, following the principles of sagittal anatomy. The surgical technique comprised three steps: cephalic, caudal dorsal, and CVL+D3. Anatomical landmarks were exposed to ensure quality control for each surgical area. Intraoperative photographs were captured, and data on operation time, lymph node harvest, intraoperative blood loss, and postoperative outcomes were collected.

**Results:**

All laparoscopic procedures were successfully completed without the need for conversion to open surgery or the occurrence of intraoperative complications. Lymph node dissection was successfully performed in all patients, and specimens were examined pathologically. The average number of lymph nodes harvested, operation time, and intraoperative blood loss were 20.25 ± 3.23, 153.36 ± 11.49 minutes, and 42.15 ± 5.82 mL, respectively. All patients were diagnosed with adenocarcinoma based on pathological examination. The 3-year overall survival rate was 78.2%.

**Conclusion:**

When viewed through the lenses of embryological development and membrane anatomy, the sagittal approach to laparoscopic radical right hemicolectomy proves to be both safe and feasible, contributing to a more standardized and regulated approach to the procedure.

## Introduction

Colon cancer (CC) is among the most common malignancies of the digestive system worldwide, ranking third in incidence and second in cancer-related mortality (1). Right-sided CC, involving the cecum, ascending colon, hepatic flexure, and the proximal two-thirds of the transverse colon, is more common than left-sided CC and generally carries a worse prognosis (2, 3).

Surgical resection remains central to colorectal cancer treatment, and laparoscopic surgery offers comparable outcomes to open procedures (4). In recent years, laparoscopic radical right hemicolectomy has become the standard procedure for managing right-sided CC. Advances in embryology, surgical anatomy, membrane anatomy, and clinical techniques have promoted the widespread adoption of complete mesocolic excision (CME) combined with D3 lymphadenectomy for right-sided CC (5, 6).

Right-sided CC is characterized by a shorter average survival time and a lower 5-year survival rate compared to left-sided CC (7). The primary cause of treatment failure is recurrence and metastasis following radical surgery. Therefore, A standardized surgical strategy is crucial to reduce recurrence and improve survival. Although the general procedural steps for laparoscopic radical right hemicolectomy are well accepted, several aspects of its clinical application remain controversial (8). These include differing interpretations of abdominal embryology, membrane anatomy, the theoretical framework of membrane-guided dissection, the extent of D3 lymphadenectomy, and the definition of the medial surgical boundary (9).

This study builds upon principles of embryology and membrane anatomy to propose a conceptual framework of sagittal anatomy in laparoscopic radical right hemicolectomy. This perspective aims to improve technical clarity and support standardization in practice.

## Methods

### Data and methodology

A retrospective review was conducted on the clinical data of 128 patients with right-sided CC who underwent right hemicolectomy at the Department of Gastrointestinal Surgery, Unit 1, The First Hospital of Putian City, Fujian Province, between December 2020 and December 2022. The cohort included 70 males and 58 females, with a mean age of 62 years. Regarding tumor TNM stages, 20 patients were classified as stage I, 48 as stage II, and 60 as stage III. All patients underwent standardized laparoscopic radical right hemicolectomy, utilizing the sagittal approach based on membrane anatomy. This study did not involve ethical issues, and informed consent from patients or their families was not required.

### Inclusion and exclusion criteria

Patients were eligible for inclusion if they met the following criteria: (1) aged 18–85 years; (2) pathologically or radiologically diagnosed with primary right-sided colon cancer involving the cecum, ascending colon, hepatic flexure, or proximal transverse colon; (3) underwent elective laparoscopic radical right hemicolectomy with curative intent using the sagittal anatomical approach between December 2020 and December 2022; and (4) had complete clinical and pathological records available. Exclusion criteria included: (1) history of previous abdominal malignancy or concurrent malignancy; (2) prior major abdominal surgery affecting mesenteric anatomy; (3) BMI > 35 kg/m^2^ or < 16 kg/m^2^; (4) emergent surgery due to bowel perforation or obstruction; and (5) incomplete follow-up or missing key intraoperative data. A total of 136 patients were initially screened. After applying the inclusion and exclusion criteria, 128 patients were included in the final analysis. Eight patients were excluded due to incomplete data (n = 5), prior extensive abdominal surgery (n = 2), or BMI exceeding 35 kg/m^2^ (n = 1).

### Sagittal anatomy and procedures for laparoscopic radical right hemicolectomy

#### Membrane anatomy

**Figure 1:**
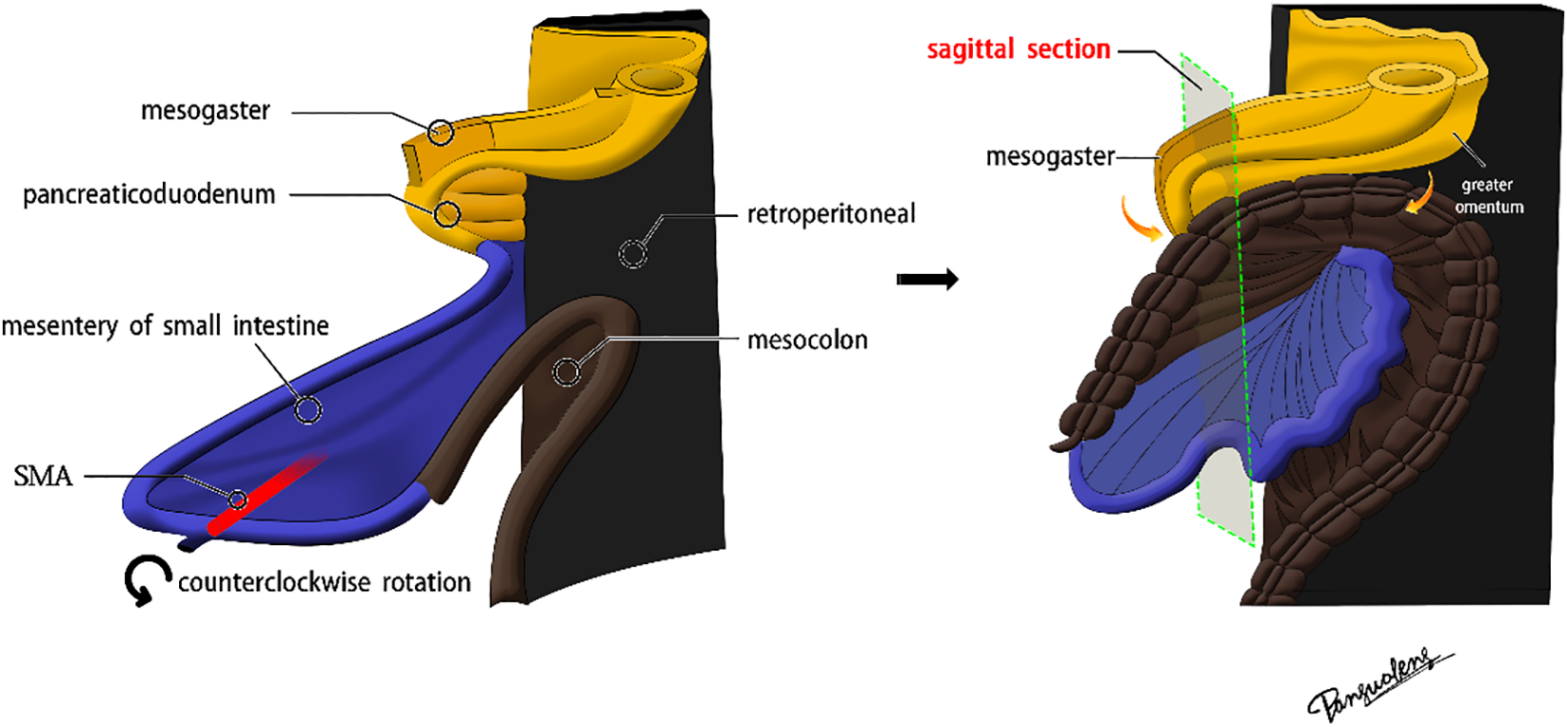
The diagram of mesogaster formation in the digestive tract.

**Figure 2:**
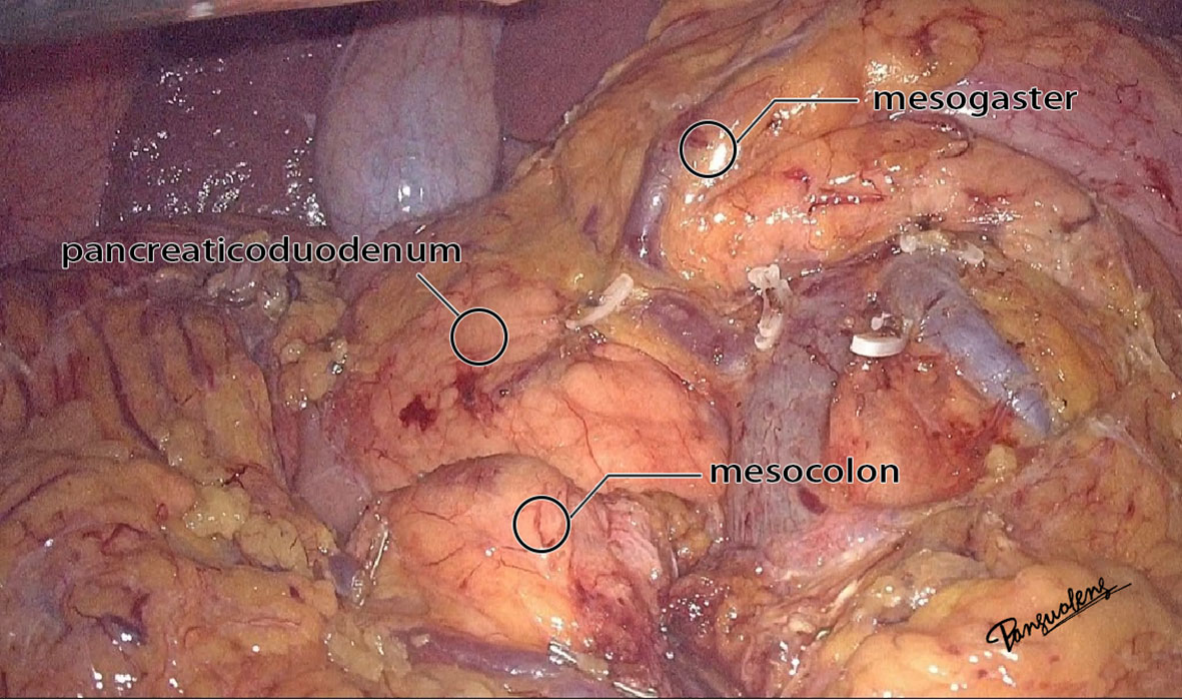
The real image of the separation of mesocolon and mesogaster in front of the pancreas head.

The superior mesenteric artery (SMA), which directly continues with the vitelline artery, serves as the axis of intestinal rotation during embryological development. A substantial counterclockwise rotation was performed on the original intestinal segment. Below the pyloric region, the mesogaster interposed between the pancreaticoduodenum and the transverse mesocolon. The greater omentum extended over the transverse colon, covering the ventral surface of the transverse mesocolon (**Figures 1**, **2**).

#### The diagram of sagittal anatomy

**Figure 3:**
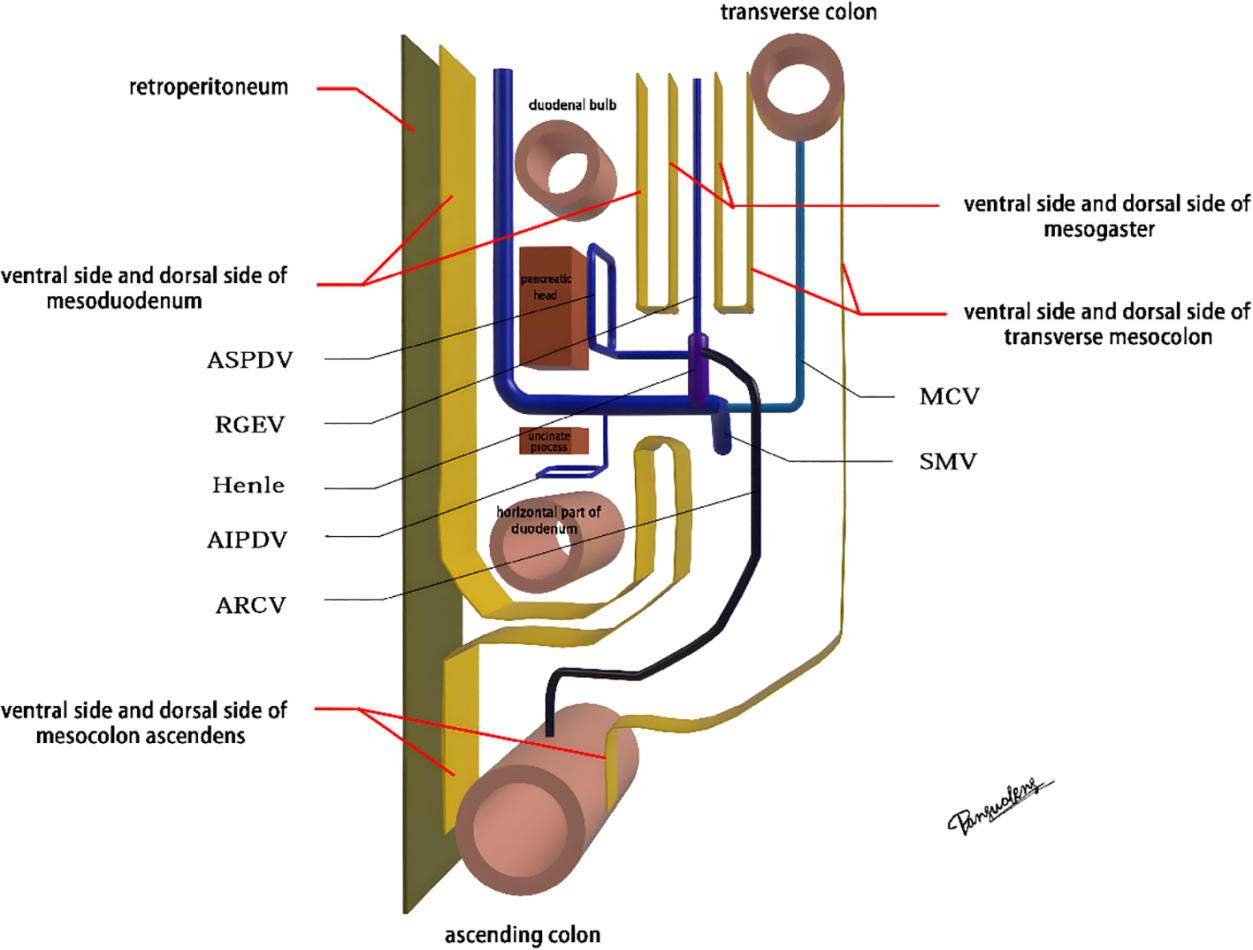
The diagram of sagittal anatomy through the Henle stem.

**Figure 4:**
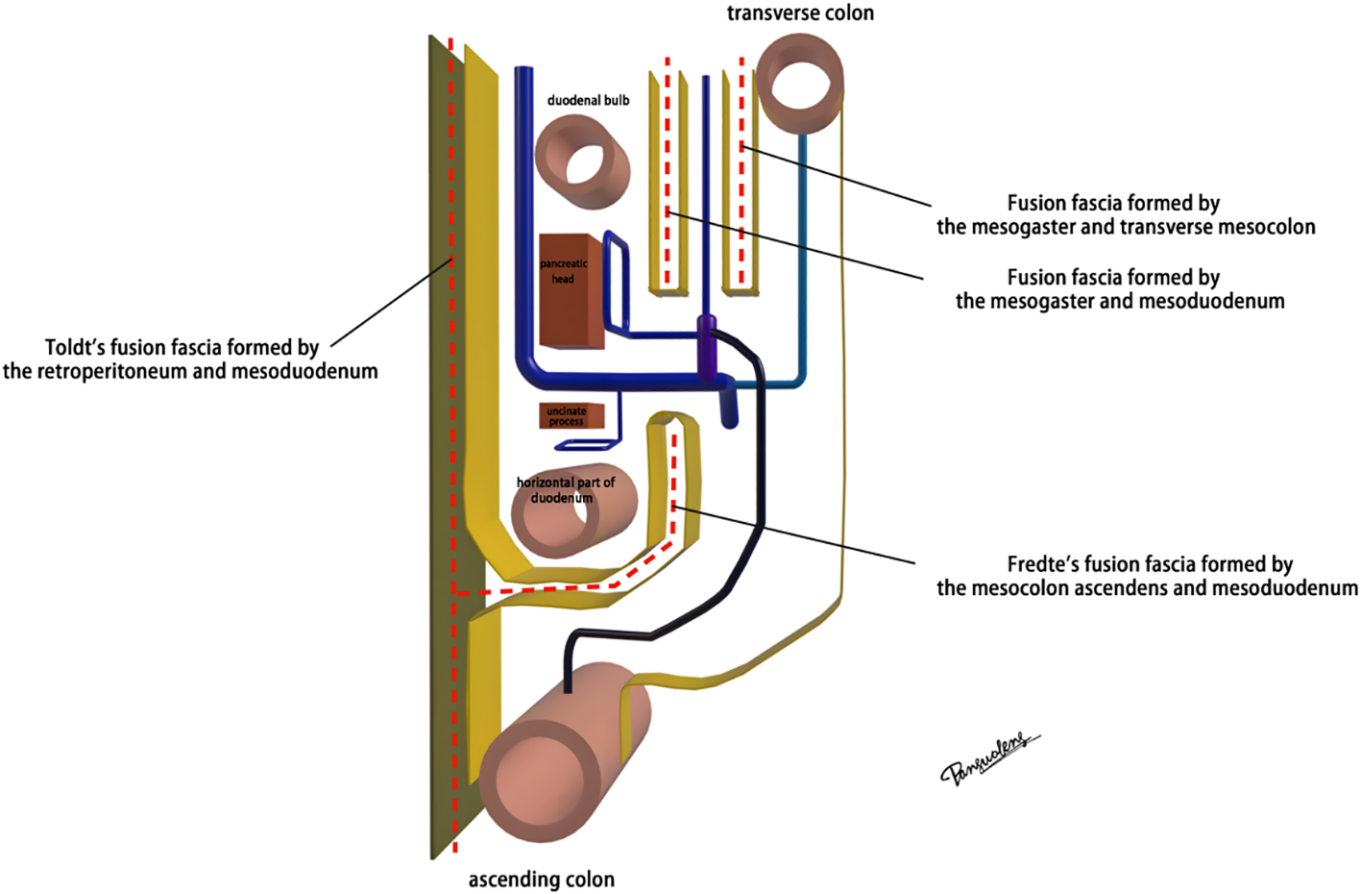
The fusion fascia formed by the right mesocolon and adjacent mesenteries.

The mesoduodenum, mesogaster, and mesocolon were apposed to form a natural avascular gap or surgical plane. The sagittal plane was defined at the Henle stem (right view, **Figure 3**). Venous drainage from the right gastroepiploic vein (mesogastrium), the anterior superior pancreaticoduodenal vein (mesoduodenum), and the accessory right colic vein in the right mesocolon converges at the Henle stem. This stem served as the common opening for the three mesenteries and the root of all three. At the confluence of the vessels at the Henle stem, both the right and left sides were fully separated, without any vascular confluence or traffic. Notably, the sagittal view was obstructed by the SMV, and the accompanying SMA and its tributaries were not visible. The core of the right colon CME surgery involved the dissection of the fusion fascia between the dorsal lobe of the right mesocolon and the two adjacent mesenteries (mesogaster and mesoduodenum). The greater omentum covering the ventral lobe of the right mesocolon was not involved in this dissection, and this section was omitted (**Figure 4**).

#### Specific steps

**Figure 5:**
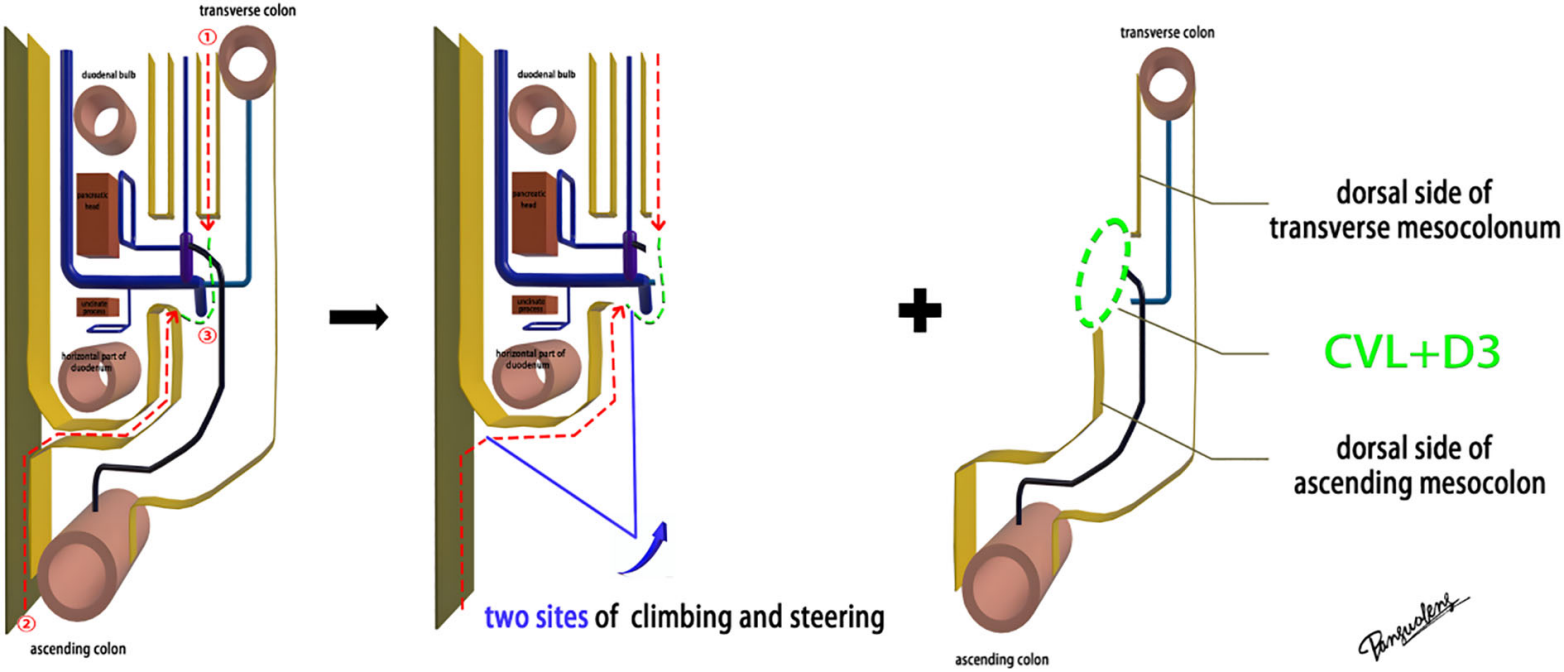
The diagram of CME surgery on the right colon in sagittal anatomy.

**Figure 6:**
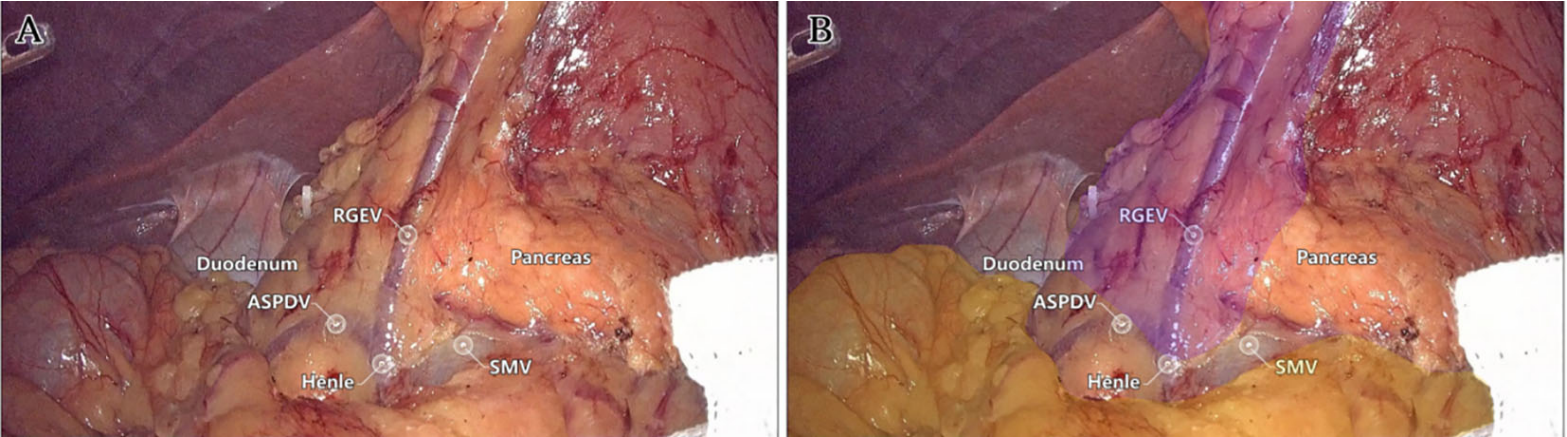
The first step: Live view of dissociation at the cephalad approach level. **(A)** The quality control criteria for completion were clear visualization of the gastrocolic venous trunk, superior anterior pancreaticoduodenal vein, and right gastroepiploic vein below the mesentery. **(B)** Mesogaster (purple) and transverse mesocolon (yellow) regions were highlighted.

**Figure 7:**
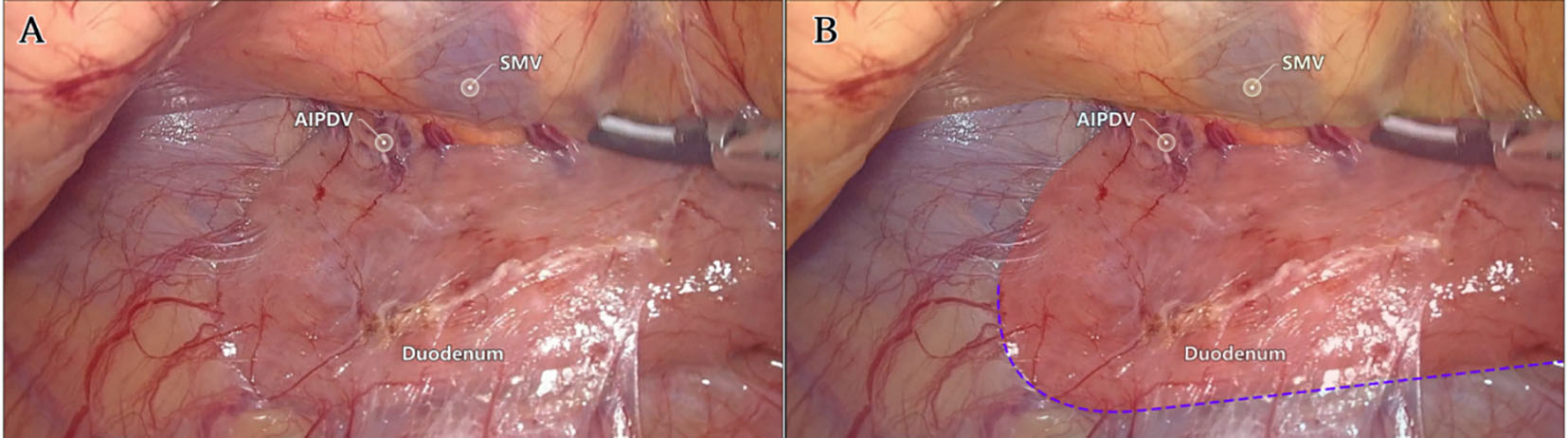
The second step: Live view of dissociation at the caudal-dorsal approach level. **(A)** The quality control criteria for completion were dorsal visualization of the uncinate process of the pancreas and pancreaticoduodenal inferior anterior vein below the ventral lobe of the mesoduodenum, and ventral visualization of the SMV below the dorsal lobe of the mesocolon ascendens. **(B)** Fredet fusion fascia (red), Toldt fusion fascia (blue), and colonic mesentery (yellow) regions were highlighted. The purple dotted line identified the first site of climbing and steering.

**Figure 8:**
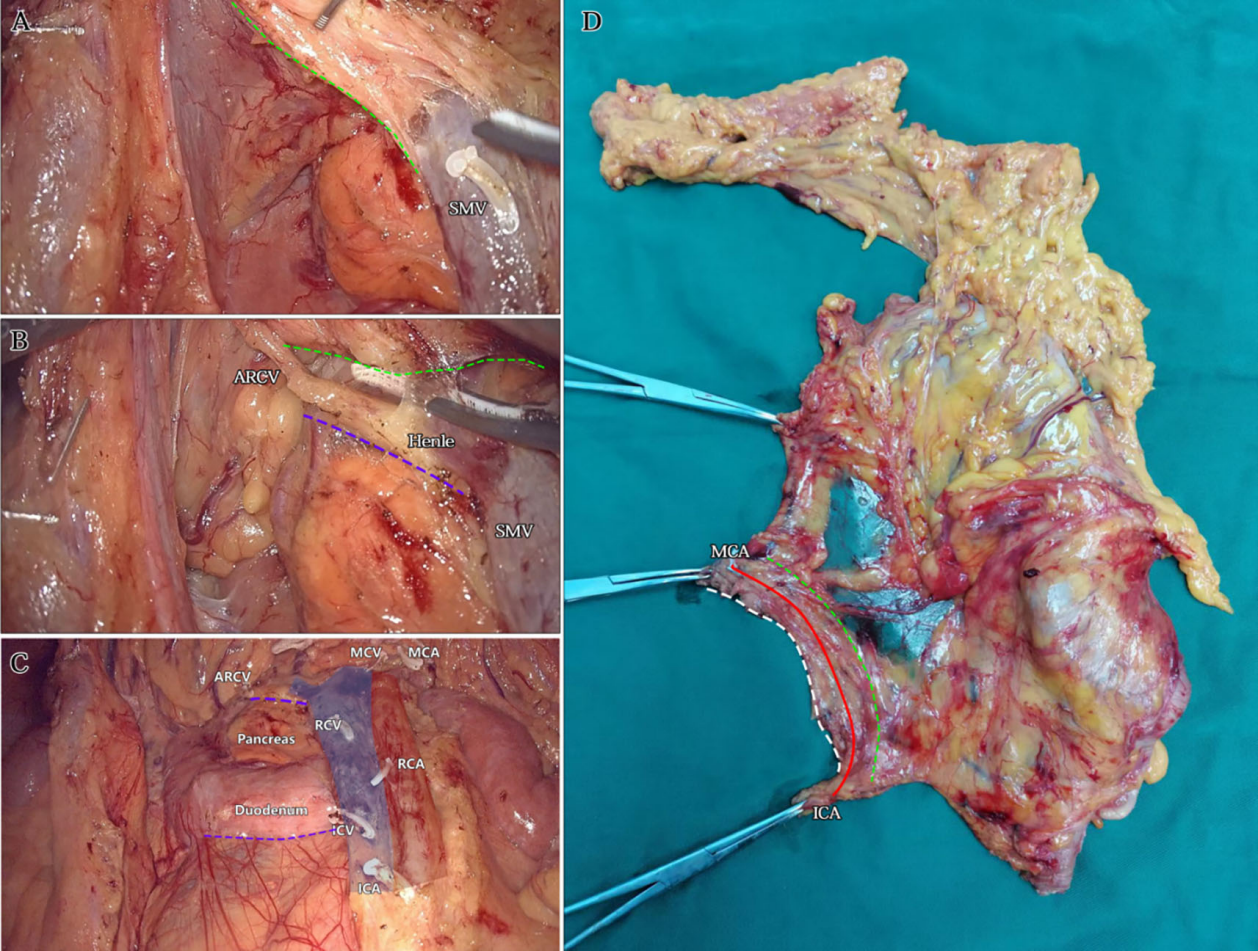
The third step: CVL+D3. **(A)** The dorsal side junction with the caudolateral approach on the right side of the SMV. The green dotted line identified the bottom edge of the dorsal lobe of the ascending mesocolon. **(B)** The ventral side junction with the caudolateral approach above the Henle stem. The green dotted line identified the bottom edge of the dorsal lobe of the transverse mesocolon. The purple dotted line identified the second site of climbing and steering. **(C)** Postoperative view after CVL and D3. The roots severed end of each innervating vessel of the right colon were identified. The purple dotted lines identified the two sites of climbing and steering. **(D)** Dorsal view of isolated specimen. The green dotted line identified the SMV vascular blot, i.e. the bottom edge of the dorsal lobes of the right colonic mesentery. The solid red line identified the line from the ICA root to the MCA root. The white dotted line identified the bottom edge of the ventral lobe of the right colonic mesentery.

Laparoscopic extended right hemicolectomy for stage T3 hepatic flexure of CC was performed, following the stepwise approach outlined in **Figure 5**. (1) Cephalad approach: The greater omentum was incised along the extramental arch to access the omental bursa and expose the pancreas. The fusion fascia between the mesogastrium and transverse mesocolon was dissected beneath the pancreatic neck, from left to right, to reveal the descending portion of the duodenum and expand the gap inferiorly. The procedure was deemed complete upon clear visualization of the gastrocolic venous trunk, the superior anterior pancreaticoduodenal vein, and the right gastroepiploic vein beneath the mesentery, as shown in **Figure 6**. Simultaneously, the dorsal lobe of the colonic mesentery was incised along Henle’s stem to expose the path converging on the SMV. The lower edge of the pancreas was mobilized to the left of the SMA, with a gauze strip placed as a guide. Notably, T3 tumors were identified as intramesenteric carcinoma, and dissection was performed through the gaps mentioned above. The No. 6 lymph node, located in the omental arch, was explored intraoperatively. If metastasis was suspected, the lymph node was cleared, indicating the necessity to enter the deeper fused fascial space between the mesogastrium and mesoduodenum. (2) Caudal-dorsal approach: The Toldt fusion fascia was dissected to the lower edge of the horizontal duodenum, moving from the anal side towards the head. Dissection continued along Fredet’s fusion fascia near Henle’s stem, with a climbing and steering maneuver to the anterior direction. To prevent deepening of the dissection, two climbing and steering sites were utilized; the first site was at this step. Completion criteria included dorsal visualization of the uncinate process of the pancreas and the inferior anterior pancreaticoduodenal vein beneath the mesoduodenum’s ventral lobe, as well as ventral visualization of the SMV beneath the dorsal lobe of the ascending mesocolon, as shown in **Figure 7**. At this stage, the entire dorsal lobe of the right colonic mesentery was fully detached from the mesenteric bed. (3) CVL and D3: Finally, we focused on the ventral aspect of the colonic mesentery, where we identified the projection of the SMA onto the mesentery. The ventral lobe of the colonic mesentery was incised along its left edge, exposing the SMA, SMV, and Henle’s stem from the bottom upwards. The ileocolic vessels, right colonic vessels, paracolic right colonic vein, and mesocolic vessels were sequentially ligated at their ventral side during root ligation. Two junctions were established: the first was located on the dorsal side with a caudolateral approach on the right side of the SMV. The second junction was created by moving below Henle’s stem, along the right side of the SMV, followed by climbing and steering upwards (the second climbing site), ultimately completing the ventral side junction with a cephalic approach. Subsequently, the guide gauze, placed during the cephalad approach, became visible. This process finalized the CVL and the clearance of the D3 lymph node in the right colonic mesentery. The quality control criterion for this procedure was the complete opening of the right colonic mesenteric envelope structure, from the ICA root to the MCA root connection. Furthermore, in the quality control of the isolated specimen, the dorsal lobe of the right colonic mesentery appeared smooth and intact, with no signs of breakage. The visibility of the SMV and SMA vascular blot, representing the bottom edge of the dorsal and ventral lobes of the right colonic mesentery, further confirmed the completeness of the mesenteric resection (**Figure 8**).

## Results

### Baseline demographic and clinical characteristics

A total of 128 patients were included in the study, with a mean age of 62.02 ± 7.53 years. The cohort comprised 70 males (54.69%) and 58 females (45.31%). The average BMI was 23.43 ± 2.11 kg/m^2^. Regarding tumor stage, 20 patients (15.63%) were stage I, 48 (37.50%) were stage II, and 60 (46.88%) were stage III. Tumor locations included the cecum (18 cases), ascending colon (44 cases), hepatic flexure (46 cases), and transverse colon (20 cases). The mean preoperative hemoglobin level was 128.62 ± 12.43 g/L, and albumin level was 38.20 ± 3.74 g/L. Comorbidities were common, with 56 patients (43.75%) having hypertension, 34 (26.56%) with diabetes mellitus, and 18 (14.06%) with cardiovascular disease. These baseline demographic and clinical characteristics are summarized in **Table 1**.

**Table 1:** Baseline demographic, clinical, and surgical outcomes of the study cohort.

Projects	Laparoscopic radical surgery was performed according to sagittal anatomical ideas
Age (years)	62.02 ± 7.53
Sex (Male/Female)	70/58
BMI (kg/m^2^)	23.43 ± 2.11
TNM stage (I/II/III)	20/48/60
Tumor location (cecum/ascending/hepatic flexure/transverse)	18/44/46/20
Preoperative hemoglobin (g/L)	128.62 ± 12.43
Preoperative albumin (g/L)	38.20 ± 3.74
Comorbidities (HTN/DM/CVD)	56/34/18
Operation time (min)	183.36 ± 15.49
The volume of intraoperative blood (ml)	42.15 ± 6.82
Number of lymph nodes cleared (pieces)	45.25 ± 7.23
Length of hospital stay after surgery (d)	9.62 ± 1.06
Length of ventilate after surgery (d)	2.26 ± 0.56

### Surgical status

All laparoscopic procedures were successfully completed without conversion to open surgery or intraoperative complications. Lymph nodes were submitted for pathological examination, and both video and photographic documentation of the procedures were obtained. Postoperative specimens were photographed for supplemental evaluation. All patients were pathologically diagnosed with adenocarcinoma based on surgical specimen analysis. The surgical and oncological outcomes for the 128 patients are summarized in **Table 1**.

### Postoperative complications and follow-up

#### Incidence of postoperative complications

Postoperative complications included 3 cases (2.34%) of incision-site infection, 0 cases of anastomotic fistula, 1 case (0.78%) of subcutaneous emphysema, 0 cases of urinary tract infection, 3 cases (2.34%) of gastroparesis, 2 cases (1.56%) of chylous fistula, 2 cases (1.56%) of adhesive intestinal obstruction, and 0 cases of bleeding. In total, 11 patients (8.59%) experienced postoperative complications.

#### Follow-up

The median follow-up duration was 25.5 months (interquartile range: 11.0–36.8 months). Ten patients (7.81%) experienced local recurrence or distant metastasis following surgery. The 3-year overall survival rate was 78.2% for the entire cohort.

## Discussion

The clinical necessity of No. 6 lymph node dissection in radical surgery for right-sided CC remains a matter of ongoing debate (10, 11). The Henle stem, an essential venous axis during embryonic intestinal rotation, represents the confluence of the mesogastrium, mesoduodenum, and the right colonic mesentery. Zheng et al. (12) suggested that lymphatic drainage from tumors at the hepatic flexure may spread via the Henle stem, reaching the junction of the mesogastrium, i.e., the No. 6 lymph node. In our view, for patients with T3 hepatic flexure tumors (i.e., intramesenteric carcinoma), routine clearance of the extra-mesenteric No. 6 lymph node may not be necessary. However, if intraoperative examination reveals lymph node enlargement with suspected metastasis, deeper tumor infiltration should be considered, warranting additional dissection of No. 6 lymph nodes within the omental vascular arch. For stage T4 patients, where cancerous lesions have infiltrated beyond the mesentery, routine dissection of No. 6 lymph nodes is indicated.

Advances in laparoscopic techniques have significantly improved intraoperative visualization of anatomical structures during radical colorectal cancer surgery. This study, grounded in laparoscopic surgical principles and informed by embryological and membrane anatomical perspectives, emphasizes the significance of the sagittal anatomy at the Henle stem. It serves to resolve the ongoing debate surrounding the posterior fascia anatomy of the transverse and ascending colon. As illustrated in **Figure 4**, the horizontal portion of the duodenum is supported by two fused fasciae: Fredet’s fascia and Toldt’s fascia, which converge posterior to the ascending colon. These fascial layers converge and thicken at the inferior margin of the horizontal portion of the duodenum. This anatomical configuration accounts for the presence of a rigid obstruction encountered when accessing the posterior gap beneath the ascending colon during Koch’s incision in the descending part of the duodenum. Professor Pan Chi from China refers to this structure as the “retroperitoneal fascia” (13). In reality, it is a tri-junctional fusion fascia formed by the retroperitoneum, the dorsal mesoduodenum, and the dorsal mesocolon ascendens. During right hemicolon CME surgery, the dorsal lobe of the colonic mesentery is detached from its mesenteric bed. Dissection is performed through distinct cephalic and caudal approaches, each necessitating meticulous separation of the corresponding fascial planes. The cephalad approach allows separation of the fusion fascia between the mesogastrium and transverse mesocolon, whereas the caudal-dorsal approach targets the inferior border of the horizontal duodenum. The dissection continues from the fusion fascia of the ascending colonic mesentery, through the posterior peritoneum, to the fusion fascia between the mesocolon ascendens and mesoduodenum (Fredet’s fascia). A second climbing maneuver in the anterior Henle stem is necessary to complete the junction and free the dorsal side of the colonic mesentery. These surgical pathways align with the findings reported in a 2022 German study of the retro-colonic fascial system (14).

When employing a basic caudal or central approach, upward dissection without dorsal anatomical guidance may result in injury to the ASPDV or AIPDV in the mesoduodenum, resulting in transmembrane dissociation due to the absence of dorsal level guidance and unclear pathways. In contrast, initiating dissection at the correct fascial plane before vascular ligation ensures complete resection of the colonic mesentery, particularly the dorsal mesentery. In contrast, performing vascular ligation prior to establishing the correct fascial plane may result in overly deep dissection in the free level (causing transmembrane dissociation) or insufficient depth in the intramembranous level, ultimately leading to mesenteric damage and incomplete resection. The technique described herein addresses these limitations by prioritizing early exposure of the dorsal mesenteric plane of the dorsal lobe of the colonic mesentery, providing a clear guide for subsequent dissection of the CVL and D3. This method also minimizes the impact of variations in bifurcation or vascular anomalies, preventing serious complications such as hemorrhage caused by errors in level determination and vascular injury. Furthermore, this technique replicates the spatial relationships between the three mesenteries (mesogastrium, mesoduodenum, and transverse mesocolon), with particular attention to the alignment of perforating vessels (e.g., ARCV within the transverse mesocolon). These vessels are identified at the surface of the Henle stem during the ventral freeing process. When dissecting the Henle stem near the pancreas, several branches of the SMV at the neck of the pancreas are visible at deeper levels, indicating the mesoduodenum’s position. At this point, further penetration should be avoided to prevent vascular injury and bleeding. Importantly, no vascular dissection was performed; only the anatomical plane was cleared, preserving the integrity of the Henle’s stem and reducing the risk of bleeding associated with the traditional cephalad approach. This method significantly reduces procedural difficulty (15). It is particularly beneficial for the surgical resection of benign right colon tumors that cannot be removed endoscopically and require direct visual dissection, especially when the right colon must be freed and loosened.

Furthermore, the standardized sagittal approach grounded in membrane anatomy may offer practical advantages across varied clinical settings. In surgical centers lacking extensive experience with CME, this method offers a reproducible anatomical framework that helps reduce technical variability and improves surgical safety. For patients with significant comorbidities who may not tolerate long operative times or significant blood loss, the clear avascular planes defined in this technique can help reduce intraoperative risks. Therefore, this approach has the potential to enhance surgical outcomes and facilitate wider adoption of high-quality radical right hemicolectomy, particularly in resource-limited or training-focused environments.

The outcomes observed in this study are generally consistent with those reported in previous studies on laparoscopic right hemicolectomy. For instance, Hohenberger et al. reported an average of 30–35 lymph nodes harvested during CME with D3 dissection (6), while our study yielded a mean of 45.25 ± 7.23 lymph nodes, which may reflect the enhanced exposure and clearance achieved via the sagittal anatomical approach. The mean operative time in our cohort (183.36 ± 15.49 min) is also comparable to findings from Wang et al. (8), who reported a mean time of approximately 180 minutes for SMA-oriented laparoscopic CME. Regarding postoperative outcomes, our complication rate (8.59%) is relatively low compared with published complication rates ranging from 10–20% in similar procedures (5, 10). Additionally, the average length of postoperative hospital stay in our cohort (9.62 ± 1.06 days) is comparable to or shorter than that reported in national colorectal surgery databases in China and Europe. These comparisons suggest that the sagittal anatomical approach is not only feasible but also yields competitive perioperative outcomes when benchmarked against established techniques.

Several limitations of this study should be considered. First, it was designed as a single-arm retrospective analysis without a control cohort or direct comparison with conventional laparoscopic radical right hemicolectomy techniques, such as the standard medial-to-lateral or caudal-to-cranial approaches. Consequently, the relative advantages of the sagittal approach in terms of surgical safety, lymph node yield, and oncological outcomes remain to be formally validated. Second, all procedures were performed at a single high-volume institution by a surgical team experienced in membrane anatomy, which may limit the generalizability of the findings to centers with different levels of technical expertise. Third, long-term oncological outcomes beyond three years were not available for all patients, and further prospective studies with extended follow-up are necessary to assess recurrence patterns and survival benefits. Fourth, this study did not include an assessment of patients’ postoperative quality of life (QoL) or functional outcomes, such as bowel function, physical recovery, or nutritional status. These metrics are increasingly considered essential for evaluating the overall effectiveness of surgical oncology procedures, particularly those involving the gastrointestinal tract. Future prospective studies should incorporate validated QoL instruments (e.g., EORTC QLQ-C30) and standardized measures of functional recovery to provide a more comprehensive understanding of patient-centered outcomes. Lastly, although the procedure demonstrated reproducibility and technical feasibility, its learning curve and broader applicability—particularly in centers without prior CME experience—warrant further investigation.

In summary, membrane anatomy provides a conceptual framework for defining the anatomical boundaries of an intact mesentery. During the dorsal mesenteric dissection, it is essential to dissociate the adjacent mesenteric fusion fascia based on the mesentery and mesenteric bed, ensuring the mesentery is separated intact from the mesenteric bed. This approach facilitates the integration of the CME and CVL/D3. The clear visualization of the SMV and SMA imprints, along with the preservation of the smooth dorsal lobe of the right colonic mesentery in specimen quality control, further supports the standardization of surgical procedures. Furthermore, we propose that the sagittal anatomical concept aligns with the primary dissection axis at the surgical level, which enhances comprehensibility for trainees and contributes to the standardization and regulation of the procedures. However, the validity and efficacy of this surgical technique must be confirmed through rigorous and well-designed clinical trials.

## Data Availability

The raw data supporting the conclusions of this article will be made available by the authors, without undue reservation.
